# Evaluation of uterine receptivity after gonadotropin releasing hormone agonist administration as an oocyte maturation trigger: a rodent model

**DOI:** 10.1038/s41598-019-48918-3

**Published:** 2019-08-29

**Authors:** Kenji Ezoe, Nana Murata, Akiko Yabuuchi, Tamotsu Kobayashi, Keiichi Kato

**Affiliations:** Kato Ladies Clinic, 7-20-3 Nishishinjuku, Shinjuku-ku, Tokyo, 160-0023 Japan

**Keywords:** Developmental biology, Embryogenesis, Intrauterine growth

## Abstract

In natural cycle or minimal stimulation cycle IVF, buserelin acetate (buserelin), a gonadotropin-releasing hormone agonist, is often used as a maturation trigger; however, its effect on pregnancy outcomes remains unclear. Therefore, in the present study, we compared uterine receptivity in buserelin-administered mice with that in human chorionic gonadotropin (hCG)-administered mice during the peri-implantation period. Implantation, decidualisation, and term-pregnancy were impaired following hCG, but not buserelin administration. hCG stimulated the synthesis and secretion of progesterone and oestradiol, whereas ovarian steroidogenesis in the buserelin-treated group was comparable with that in the control group. Furthermore, similar to the observation in controls, the buserelin-treated group exhibited activation of progesterone receptor signalling and inhibition of oestrogen receptor signalling in the endometrial epithelium on the day of implantation. However, epithelial progesterone signalling was not detected, and a high expression of genes downstream to oestrogen was observed on day 4 following hCG administration. These results suggest that buserelin administration does not impact uterine receptivity as it did not affect ovarian steroidogenesis and endometrial steroid signalling. Therefore, buserelin is preferred as an oocyte maturation trigger to optimise uterine receptivity during treatments involving timed intercourse, intrauterine insemination, or fresh embryo transfer following *in vitro* fertilisation.

## Introduction

During the 1990s, gonadotropin releasing hormone (GnRH) agonists were introduced as an alternative to human chorionic gonadotropin (hCG) for inducing oocyte maturation^1–4^. Historically, hCG administration had been the primary method to induce oocyte maturation in fertility treatments, including timed intercourse, intrauterine insemination, and *in vitro* fertilisation (IVF). When administered in cycles coinciding with multiple follicular development, hCG triggers the formation of multiple corpora lutea while stimulating luteal function and generating supraphysiological oestradiol and progesterone levels^5^. In contrast to hCG, which induces an increase in only luteinising hormone (LH)-like activity, GnRH agonists activate GnRH receptors in the pituitary, inducing surges in both LH and follicle stimulating hormone (FSH)^3,6,7^. These surges promote nuclear maturation and corpus luteum formation^6,8,9^, similar to the natural cycle^2,7^. In addition, GnRH agonists have lower carbohydrate content than hCG, resulting in a shorter circulating half-life and reduced duration of LH receptor activity^10–13^. These characteristics lower the risk of ovarian hyperstimulation syndrome (OHSS)^14–18^. Furthermore, hCG exerts negative effects on endometrial receptivity^19–22^ and embryo quality^23^.

These disadvantages of hCG treatment are a major reason for the interest in GnRH agonists. Of these, buserelin appears to generate significantly more mature oocytes than hCG following ovulation induction^5^. However, buserelin also significantly lowers implantation and clinical pregnancy rates, while increasing early pregnancy loss. These negative outcomes are not universal as a previous study failed to find adverse effects on implantation or pregnancy after oocyte maturation was induced^24^. Unfortunately, few studies have compared pregnancy outcomes after hCG or GnRH-agonist administration, causing difficulties in determining which treatment is effective while minimising side effects. Thus, in the present study, we examined the effects of buserelin administration on implantation, decidualisation, and term pregnancy in mice. We also assessed effects on ovarian steroidogenesis and endometrial steroid signalling, as both processes are crucial for uterine receptivity.

## Results

### Buserelin did not impair implantation, decidualisation, and term pregnancy

**Figure 1:**
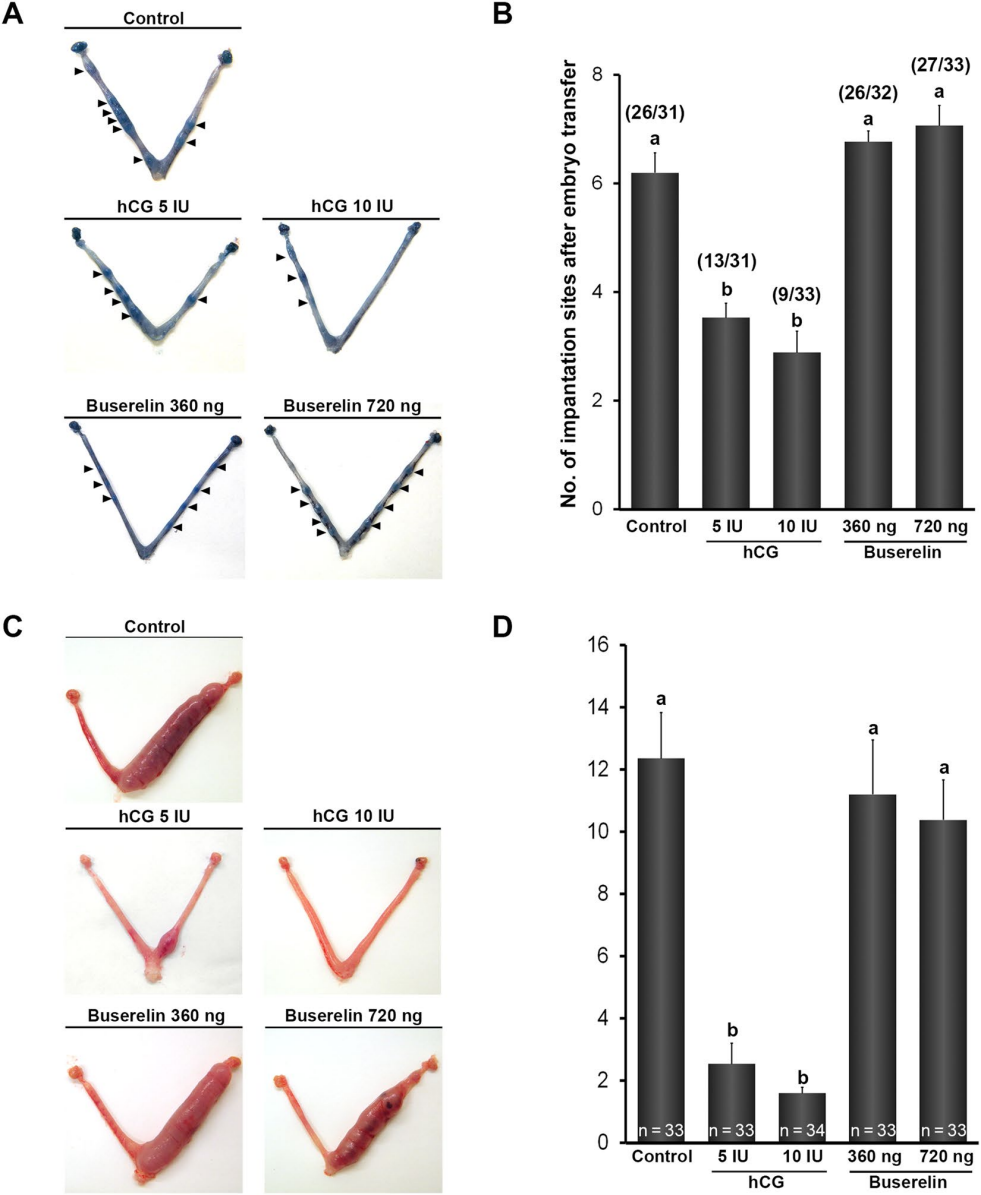
Embryo implantation and formation of decidual tissue after hCG or buserelin administration in mice. (**A**) Representative uteri on day 5 of pregnancy. Arrowheads indicate implantation sites. (**B**) Number of implantation sites on day 5 of pregnancy. Ten blastocysts were transferred to uteri on day 4 of pseudopregnancy, and the number of implantation sites was assessed on day 5. Values above bars indicate the amount of pregnant mice over total number of mice. (**C**) Decidualisation in hyper-stimulated mice after intrauterine oil infusion of uterine right horn (left horn was control). (**D**) Fold-increase in uterine weight. Decidualisation extent was calculated as fold-change in uterine weight of oil-infused horns divided by change in non-infused horns. Values above bars indicate number of mice with decidualisation over total number of mice. Error bars represent standard error of the mean. Bars labelled with distinct letters are significantly different from each other (*P* < 0.05).

To evaluate the uterine receptivity after administration of hCG or buserelin, blastocyst stage embryos were transferred to pseudo-pregnant mice that were primed with either hCG or buserelin, and analysis of blastocyst implantation on day 5 was performed. In the saline injected (control) group, we observed implantation sites (average number: 6.3 ± 0.4) in 83.9% of female mice after embryo transfer (Fig. 1A,B). We also observed a dose-dependent reduction in the number of mice with implantation sites (5 IU: 41.9%, 10 IU: 27.3%) and in the number of implanted blastocysts (3.5 ± 1.0, 2.9 ± 1.2; respectively) in hCG-treated groups. However, buserelin groups did not differ from control in terms of the percentage of mice with implantation sites (360 ng: 81.3%, 720 ng: 81.8%) and the number of implantation sites (6.8 ± 0.2, 6.9 ± 0.4, respectively).

To determine the decidualisation ability in endometrium post implantation period, artificial decidualisation was induced by oil infusion into the uterine horn of hCG or buserelin treated pseudo-pregnant mice. Regardless of dosage, endometrial decidualisation was compromised in hCG-treated groups (5 IU: 2.5 ± 0.7, 10 IU: 1.6 ± 0.2) compared with that in control (12.4 ± 1.5) (Fig. 1C,D). By contrast, fold increase in uterine weight was similar across control and buserelin-treated groups (360 ng: 11.2 ± 1.8, 720 ng: 10.4 ± 1.3).

Furthermore, the effect of hCG and buserelin administration on post-implantation development and term pregnancy was examined by embryo transfer. Compared with the control group, hCG-treated groups had significantly lower pregnancy rates after blastocyst transfer (5 IU: *P* = 0.0004, 10 IU: *P* < 0.0001, Table 1), whereas rates in buserelin-treated groups did not differ (360 ng: *P* = 0.2482, 720 ng: *P* = 0.1570). Pup number and weight were also considerably lower among hCG-treated mice than among the control. Buserelin treatment did not result in significantly fewer pups than control, although there was a tendency toward lower pup rate in the former group (360 ng: *P* = 0.0706, 720 ng: *P* = 0.0595). Together, these results indicate that hCG hampered implantation and decidualisation more so than did buserelin, leading to decreased pregnancy rates.

**Table 1:** Post-implantation embryonic development after blastocyst transfer into pseudopregnant mice (artificially induced ovulation through mating with vasectomised males).

Experimental group	No. of recipient mice	No. of full-term pregnant mice (%)	No. of transferred blastocysts	No. of pups (%)	Pup weight (g)	Placental weight (g)
Control	32	26 (81.3)^a^	384	149 (38.8)^a^	1.286 ± 0.031^a^	0.144 ± 0.002
hCG, 5 IU	32	12 (37.5)^b^	384	49 (12.8)^b^	0.965 ± 0.028^b^	0.142 ± 0.005
hCG, 10 IU	33	11 (33.3)^b^	396	47 (11.9)^b^	0.817 ± 0.057^b^	0.138 ± 0.006
Buserelin, 360 ng	32	22 (68.8)^a^	384	125 (32.6)^a^	1.270 ± 0.037^a^	0.140 ± 0.003
Buserelin, 720 ng	32	21 (65.6)^a^	384	124 (32.3)^a^	1.287 ± 0.022^a^	0.135 ± 0.002

^a^Data in each column with distinct superscripts (a–c) are significantly different from each other (*P* < 0.05). hCG, human chorionic gonadotropin.

### Buserelin did not alter progesterone/oestradiol synthesis and secretion

**Figure 2:**
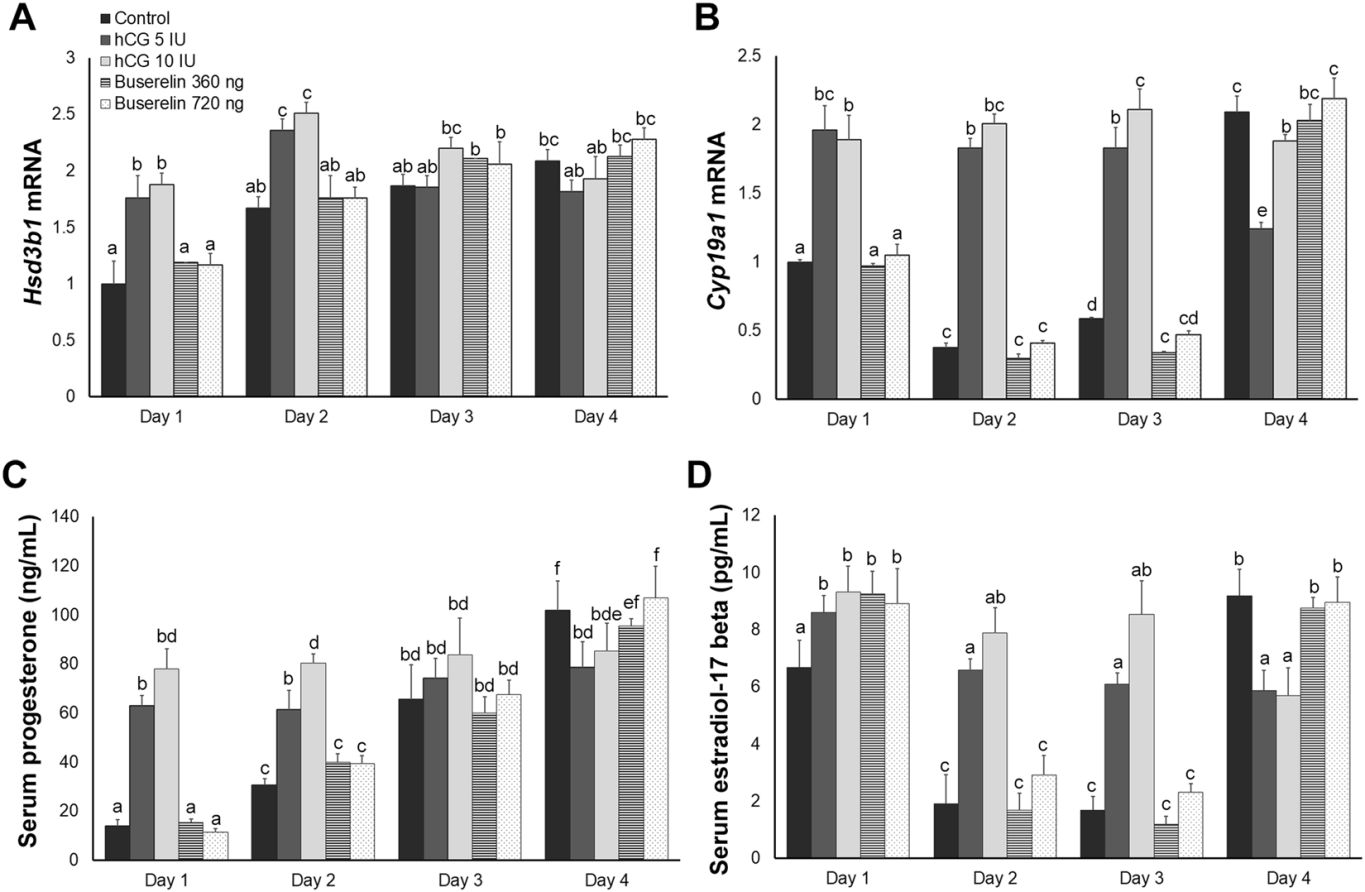
Ovarian steroid-hormone synthesis and secretion during the pre-implantation period. (**A**) *Hsd3b1* and (**B**) *Cyp19a1* mRNA expression. Serum (**C**) progesterone and (**D**) oestradiol-17β levels. Error bars represent standard error of the mean. Bars labelled with distinct letters are significantly different from each other (*P* < 0.05).

As a marker of progesterone synthesis, *Hsd3b1* is responsible for transforming pregnenolone to progesterone. This gene was upregulated on day 3 in the control and buserelin-treated groups, but on day 1 in the hCG-treated groups (Fig. 2A). *Cyp19a1* catalyses the conversion of testosterone to oestradiol; in the control group, its mRNA expression was high on day 1, then decreased on day 2 before increasing again on day 4 (Fig. 2B). Expression patterns were similar in the control and buserelin-treated groups, but higher on days 1–3 in hCG-treated groups.

Serum progesterone level increased starting from day 2 in the control and buserelin-groups (Fig. 2C). However, progesterone levels were already higher in the hCG-treated groups than in the control group on days 1–2, and levels were significantly lower than those in the control group on day 4. Control serum oestradiol decreased on day 2 before increasing again on day 4 (Fig. 2D). The hCG groups had notably higher oestradiol levels on days 1–3 before experiencing significant downregulation compared with those in the control on day 4. Buserelin-treated groups exhibited significantly higher oestradiol levels than control on day 1, but then did not differ from control on days 2–4. These results indicate that hCG dysregulated steroidogenic enzyme expression in ovaries through artificially inducing oocyte maturation. By contrast, buserelin administration did not affect the synthesis or secretion of ovarian steroid hormones during the pre-implantation period.

### Buserelin does not adversely affect PR expression and downstream signalling

**Figure 3:**
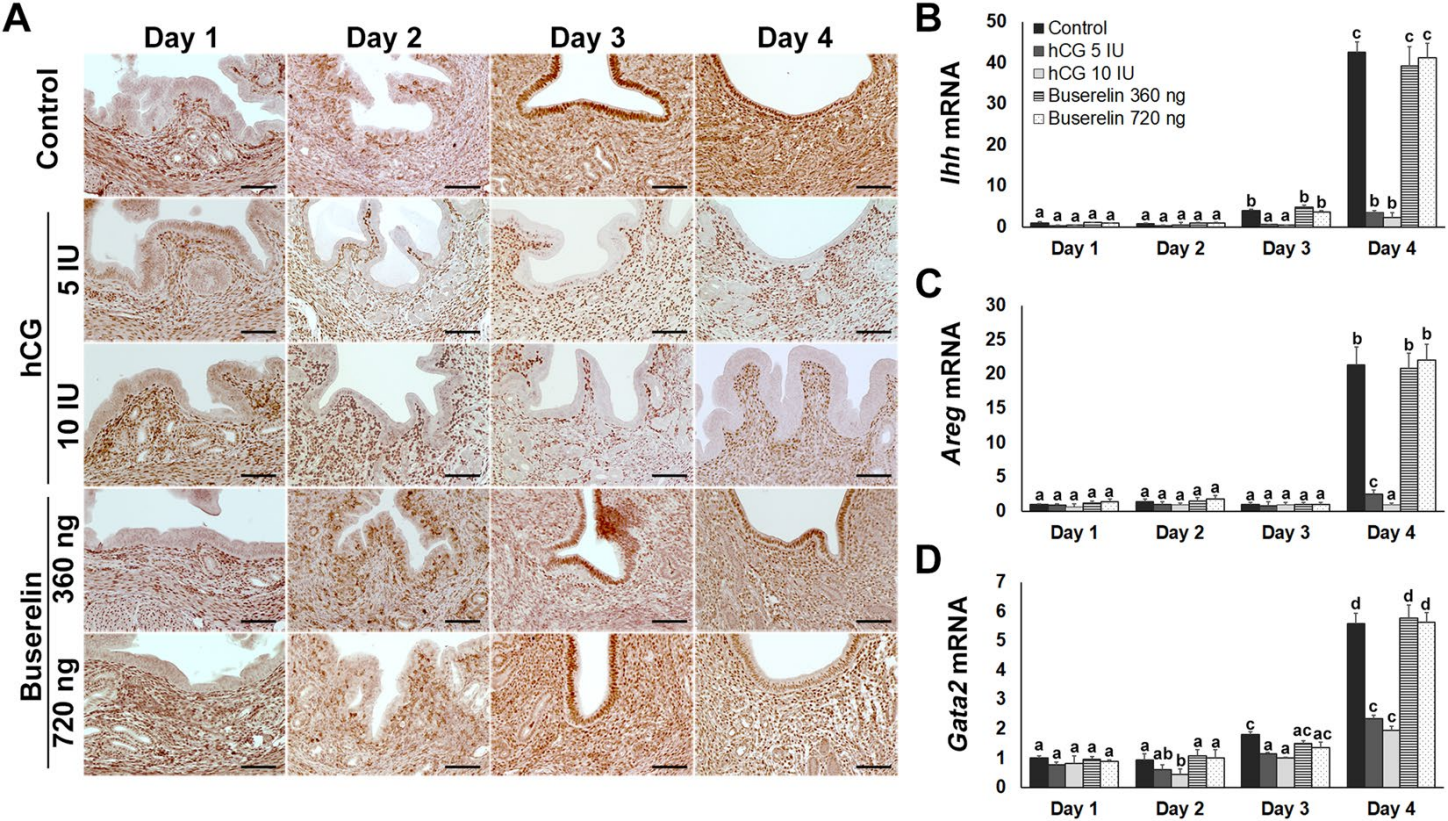
Endometrial PR signalling following oocyte maturation trigger. (**A**) Distribution of PR in endometrium during pre-implantation. Scale bars represent 100 μm. (**B**) *Ihh*, (**C**) *Areg*, and (**D**) *Gata2* mRNA expression during pre-implantation. Error bars represent standard error of the mean. Bars labelled with distinct letters are significantly different from each other (*P* < 0.05).

The expression of progesterone receptor (PR) and its downstream genes is associated with uterine receptivity in the endometrium during the pre-implantation period. In the control group, PR was detected in stromal cells from day 1 onward, as well as in the luminal epithelium on days 3 and 4 (Fig. 3A). Buserelin groups exhibited the same pattern. In contrast, hCG groups exhibited PR expression in stromal cells from day 1 onward, but not in the luminal epithelium. Thus, hCG, but not buserelin, altered the localisation of PR expression during pre-implantation, especially in luminal epithelium.

The *indian hedgehog* (*Ihh*) acts downstream of epithelial PR^25,26^ and was expressed at low levels in all groups on days 1–3. Although *Ihh* expression notably increased on day 4 in the control and buserelin-treated groups, hCG groups exhibited lower *Ihh* expression than control (Fig. 3B). We further examined mRNA expression of *amphiregulin* (*Areg*)^27,28^ and *GATA binding protein 2* (*Gata2*)^29^, two other genes downstream of epithelial PR. Both had substantially lower expression in the hCG-treated groups than in the control or buserelin-treated groups on day 4 (Fig. 3C,D). Therefore, hCG—but not buserelin—caused steroidogenic dysregulation that altered the expression of epithelial PR and its downstream genes.

### Epithelial oestrogenic signalling was not adversely affected by buserelin administration

**Figure 4:**
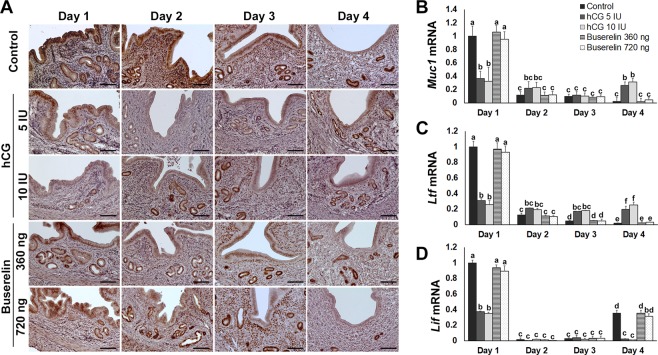
Endometrial ER signalling following oocyte maturation trigger. (**A**) Distribution of ERα in endometrium during pre-implantation. Scale bars represent 100 μm. (**B**) *Muc1*, (**C**) *Ltf*, and (**D**) *Lif* mRNA expression during pre-implantation. Error bars represent standard error of the mean. Bars labelled with distinct letters are significantly different from each other (*P* < 0.05).

Immunohistochemistry detected oestrogen receptor (ER)α in the luminal epithelium of control mice on days 1–3, then tracked a substantial decrease on day 4 (Fig. 4A). Buserelin-treated groups exhibited similar ERα expression patterns throughout the pre-implantation period. However, some endometrium epithelial cells in the hCG-treated groups exhibited low ERα expression on days 1–4.

*Mucin 1* (*Muc1*) and *lactotransferrin* (*Ltf*) are both well-characterised genes that act downstream of epithelial ERα and are typically downregulated during the implantation period^30–32^. In the control and buserelin groups, *Muc1* mRNA expression was detected on day 1, followed by a decrease in expression from day 2 onward (Fig. 4B). However, in hCG-treated groups, *Muc1* mRNA expression was low on days 1–4. Immunohistochemical analysis showed that on day 4, MUC1 protein expression decreased on day 4 in all control and buserelin-treated mice, but only in 30.8% of hCG-treated mice (Supplemental Fig. 2A,B). High *Ltf* mRNA expression was also detected on day 1, but the level markedly decreased on days 2–4 in control and buserelin-treated groups, whereas it remained detectable on day 4 in the hCG-treated groups (Fig. 4C). *Leukaemia inhibitory factor* (*Lif*) is essential for uterine receptivity and implantation^33,34^. In control and buserelin groups, *Lif* mRNA expression was high on day 1, decreased on day 2, and increased again on day 4 (Fig. 4D). In hCG groups, however, *Lif* expression was low on day 4 (Fig. 4D).

### Endometrial proliferation-differentiation switching was normally regulated after buserelin administration

Immunostaining revealed Ki67 expression in the luminal epithelium on day 1 for control and buserelin groups. Subsequently, the expression shifted to the stroma and increased on day 4 (Supplemental Fig. 2C,D). Similar to that in the control, the luminal epithelium in hCG-treated groups exhibited Ki67-positive cells on day 1. However, Ki67-positive cell localisation did not shift to the stroma and remained detectable in the luminal epithelium on day 4. These results suggest that buserelin, but not hCG, successfully inhibited estrogenic activity in the luminal epithelium during pre-implantation.

## Discussion

This is the first report to compare the effects of hCG and buserelin-acetate-induced oocyte maturation on endometrial steroid signalling, a process crucial for uterine receptivity. We first determined that hCG administration substantially impaired blastocyst implantation in the endometrium, differentiation of endometrial stromal tissues to decidua, pregnancy rates, pup numbers, and pup weight in mice. Previous studies reported that ovarian stimulation using a combination of hCG and equine CG (eCG) reduced the number of embryos in the late stage of gestation that resulted in a lower number of full-term pups in rodent models. Furthermore, embryonic and foetal development was delayed after ovarian stimulation, and the formation of pinopodes was also affected^35–37^. These adverse effects have also been observed in mice treated with hCG alone, which had reduced number of pups due to failure in either implantation or early pregnancy^21,38–40^. Our results confirmed these previous findings that hCG administration impaired pregnancy outcomes after embryo transfer. Human chorionic gonadotropin acts in conjunction with LH through the hCG/LH receptor. However, LH has a far shorter circulating half-life (25–60 min)^11,41^ than hCG (24–37 h)^12,42^. Moreover, hCG remains detectable in serum and urine for 7–11 d after injection^13^. These differing biochemical characteristics of the two hormones suggest that hCG administration will affect the implantation window (4 d post-LH surge in mouse)^43,44^, but not buserelin administration.

Next, we examined the sustained effect of hCG and buserelin on ovarian steroidogenesis during the pre-implantation period. The secretion of FSH and LH from the pituitary regulates progesterone and oestrogen, both of which are essential for implantation initiation and optimal decidualisation^44^. In hCG-treated but not in buserelin-treated or control groups, we observed aberrant expression of *Hsd3b1* and *Cyp19a1*, which are markers of progesterone and oestradiol production, respectively. Control and buserelin-treated groups also did not differ in progesterone and oestradiol secretion patterns, but hCG treatment disrupted their secretion. Therefore, hCG appears to exert prolonged effects on ovarian steroidogenesis during the implantation period, in line with a previous study demonstrating that this hormone stimulates progesterone/oestradiol production in luteal cells^45^. While buserelin elevated serum oestradiol on day 1, prolonged changes in ovarian steroidogenesis were not observed. Again, this difference in outcome is attributable to the shorter half-life of LH.

Progesterone and oestrogen regulate PR and ERα spatiotemporal expression in the uterus^46–48^. Critical for establishing and maintaining pregnancy^49,50^, PR is transiently expressed in luminal epithelium during the implantation period^51,52^. We confirmed this expression in control and buserelin-treated groups, but expression was inhibited throughout pre-implantation in hCG-treated groups. We further demonstrated that hCG administration inhibited *Ihh*, *Areg*, and *Gata2* expression. These epithelial PR target genes play critical roles during initial implantation, regulating epithelial oestrogen signalling and the cell cycle^26,53–56^. In addition, *Muc1*^32,57,58^ and *Ltf*^30,31^ epithelial oestrogen signalling remained high under hCG treatment on day 4, even though the process is normally inhibited during implantation, as observed in control and buserelin-treated groups.

Oestrogen-ER signalling promotes endometrial proliferation-differentiation switching, a marker of uterine receptivity^59^, while stromal PR signalling inhibits the process. Stromal proliferation is initiated on day 3 to prepare the uterus for implantation and decidualisation^47,59–62^. Furthermore, epithelial PR activity is essential for establishing uterine epithelial-stromal crosstalk and implantation^63^, but elevated oestrogen during pre-implantation inhibits PR^64^. We did not observe endometrial proliferation-differentiation switching in hCG-treated groups. Therefore, we speculate that when hCG stimulates oestradiol synthesis and secretion, PR signalling is inhibited, while ER signalling is stimulated in the luminal epithelium. In contrast, buserelin exerts no such effects, given that treatment with the GnRH agonist does not result in progesterone and oestradiol secretion patterns that differ from those of the control group. Our results demonstrate that buserelin administration is a method to trigger oocyte maturation that does not affect implantation, decidualisation, or subsequent term pregnancy. The drug does not affect ovarian steroidogenesis or endometrial steroid signalling during the pre-implantation period, whereas hCG clearly impaired uterine receptivity and pregnancy outcomes.

A previous study reported that the implantation process can be induced by exogenous LIF instead of oestrogen. Exogenous LIF induced implantation-associated events in the endometrium, such as closure of the uterine lumen, stromal decidualisation, and apoptosis of the luminal epithelium at the site of blastocyst attachment^65^. Therefore, we hypothesised that exogenous LIF administration could rescue the impairment of blastocyst implantation caused by hCG administration, as *Lif* expression was reduced in hCG treated mice. However, in contrast to our expectation, blastocyst implantation was not restored by LIF administration, although a slight improvement was observed in endometrial steroid signalling after the administration (Supplemental Fig. 3). Furthermore, we assessed the uterine receptivity after dual trigger with buserelin and hCG. Recent studies reported that dual trigger with GnRH agonist and hCG improved pregnancy outcomes compared to trigger with hCG only^66,67^. Although the effectiveness of dual trigger on uterine receptivity is still controversial as most recent study by Zhou *et al*. demonstrated that there are no differences in pregnancy outcomes between the dual and hCG only triggers^68^. Our results demonstrated that the dual trigger with low dose of buserelin and hCG tended to improve blastocyst implantation and endometrial steroid signalling (Supplemental Fig. 3), suggesting that the adverse effects of hCG administration on uterine receptivity may be dose-dependent and that the combination use of buserelin and low dose of hCG might be more likely to result in pregnancy outcomes after fresh embryo transfers.

While these results are promising, we note that human trials will be necessary to confirm whether the effects are carried over from the mouse model. Nevertheless, oestrogen and progesterone regulate uterine receptivity in both mice and humans^43,69^. Therefore, it is highly likely that hCG-induced hypersecretion of ovarian oestrogen and progesterone will alter epithelial PR and ER signalling in the endometrium and that these adverse effects are irreversible regardless of the treatment applied (whether recombinant LIF or dual trigger with buserelin). Our findings indicate that buserelin is preferable to hCG as an oocyte maturation trigger that can optimise uterine receptivity during fertility treatments.

## Methods

### Animals

ICR mice were obtained from Japan SLC Inc. (Shizuoka, Japan) and housed in a 12-h light/dark cycle. To induce oocyte maturation, 8-week-old female mice were divided into six treatment groups: 5 IU hCG, 10 IU hCG (Mochida Pharmaceutical, Tokyo, Japan), 360 ng buserelin acetate, 720 ng buserelin acetate (Sigma-Aldrich, St. Louis, MO, USA), and the low and moderate dose of dual trigger (low dose group, 2.5 IU hCG and 180 ng buserelin; moderate dose group, 5 IU hCG and 360 ng buserelin). Mice were injected intraperitoneally with the appropriate drug on the day of oestrus, as previously reported^21,70^, while a separate group of control mice were injected with saline. After injection, female mice were mated with 10–12-week-old vasectomised or intact ICR males to induce pseudopregnancy or pregnancy, respectively (day 1 = vaginal plug). Pseudopregnant females were subjected to the evaluation of decidualisation, as well as foetal development and implantation after embryo transfer. Pregnant females were evaluated for ovarian hormone synthesis and secretion, along with the expression of uterine steroid receptors and their downstream signalling. In some instances, 5 µg of recombinant LIF (Millipore, Burlington, MA, USA) was intraperitoneally injected into hCG-treated mice at 1600 h on days 3 and 4 of pregnancy or pseudopregnancy. All mouse work was conducted in accordance with the guideline of the institutional animal care and use committee of Kato Ladies Clinic which is based on Animal Welfare Act Regulations and the Guide for the Care and Use of Laboratory Animals. All animal experiments in this study were reviewed and approved by the institutional animal care and use committee of Kato Ladies Clinic (K011). All efforts were made to minimise the number of animals used and their suffering.

### Embryo culture and transfer

Two-cell embryos were collected from the excised oviducts of donor mice on day 2 of pregnancy and cultured for 72 h until the blastocyst stage. The culturing medium was KSOM (potassium simplex optimised medium; Millipore, Billerica, MA, USA), and conditions were 37 °C in a 5% CO_2_/95% air atmosphere. Embryo transfers and caesarean sections were performed in all groups, as described previously^21^, to evaluate full-term development. Briefly, 12 blastocysts were transferred into uteri of pseudopregnant mice on day 4. On day 19, live foetuses were removed through caesarean sections to determine pregnancy rate.

### Analysis of blastocyst implantation

Ten blastocysts were transferred into the uteri of pseudopregnant females on the fourth day after pregnancy initiation. On day 5, Chicago blue dye solution was intravenously injected into these pseudopregnant females or into naturally mated females^71^. The dye allowed for visual evaluation of implantation sites.

### Induction of artificial decidualisation

Artificial decidualisation was induced as previously described^72^. Briefly, pseudopregnancy was induced in female mice through mating with vasectomised males. Sesame oil (25 µL) was infused into one side of the uterine horn to cause decidualisation. Wet weights of infused and non-infused uterine horns were recorded on day 8. Fold induction in uterine wet weights was used as the index for comparing artificial decidualisation.

### Measurement of serum progesterone and oestradiol-17β

Serum was collected from 500 µL of blood per mouse. Serum progesterone and oestradiol levels were measured with enzyme immunoassay EIA kits (Cayman Chemical Company, Ann Arbor, MI, USA), following the manufacturer’s protocol.

### Immunohistochemistry

Uterine sections were immunostained for PR, ERα, Muc1, Ki67, and pSTAT3 using previously described procedures with slight modifications^21^. Uterine horns were collected from female mice on days 1–4 of pregnancy and fixed in 10% neutral-buffered formalin to generate paraffin sections. Sections were deparaffinised in xylene, rehydrated using a graded ethanol series, and then blocked with 3% bovine serum albumin in Ca^2+^/Mg^2+^-free Dulbecco’s phosphate-buffered saline (PBS). Sections were incubated overnight at 4 °C with the following primary antibodies: mouse anti-PR antibody (1:300, LS-B5236; LifeSpan Biosciences, Seattle, WA, USA), rabbit anti-ERα antibody (1:300, sc-7207; Santa Cruz Biotechnology, Dallas, TX, USA), rabbit anti-Muc1 antibody (1:200, NB-120-15481; Novus Biologicals, Littleton, CO, USA), rabbit anti-Ki67 antibody (1:200, RM-9106; Thermo Scientific, Waltham, MA, USA), and rabbit anti-pSTAT3 antibody (1:200, ab76315; Abcam, Cambridge, UK). Sections were washed and then incubated with the following secondary antibodies: biotinylated goat anti-rabbit antibody (1:500, 111-065; Jackson ImmunoResearch, West Grove, PA, USA) to detect ERα, Muc1, Ki67, and pSTAT3, and biotinylated goat anti-mouse antibody (1:500, 62–6540; Life Technologies, Waltham, MA, USA) to detect PR. Next, sections were incubated with horseradish peroxidase-conjugated streptavidin (1:300, 43–4323; Life Technologies) and then with chromogen 3,3′-diaminobenzidine tetrahydrochloride (DAB, 00–2007; Life Technologies) to visualise antibody staining. Finally, sections were counterstained with haematoxylin to reveal immunoreaction sites (reddish deposits). A semiquantitative approach was used to evaluate Ki67 expression. Specifically, staining intensity (SI) per cell was classified as 0–3 (Supplemental Fig. 1) for calculating an H-score from the following formula: [1 × (% of cells with SI = 1) + 2 × (% of cells with SI = 2) + 3 × (% of cells with SI = 3)].

### Quantitative RT-PCR analysis

Quantitative (q)RT-PCR assays were performed as previously described^21,73^. Ovaries and uterine horns were collected and stored at −80 °C until use. Total RNA was extracted using TRIzol reagent (Invitrogen, Carlsbad, CA, USA) and then further purified with the PureLink RNA mini kit (Life Technologies). Reverse transcription of RNA (5 μg) was performed using a high-capacity RNA-to-cDNA kit (Life Technologies). Next, mRNA levels of the following genes were quantified with TaqMan Gene Expression Assay (Life Technologies): *Hsd3b1* (Mm01261921_mH), *Cyp19a1* (Mm00484049_m1), *Ihh* (Mm00439613_m1), *Areg* (Mm01354339_m1), *Gata2* (Mm00492301_m1), *Lif* (Mm00434762_g1), *Muc1* (Mm00449604_m1), *Ltf* (Mm00434787_m1), and *Foxa2* (Mm01976556_s1). The reaction mixture (20 μL) included the TaqMan Fast Universal PCR Master Mix (4352042; Life Technologies) and sequences were amplified in a StepOnePlus Real-time PCR System (Applied Biosystems, Foster City, CA, USA). Each sample was assayed in duplicate. Glyceraldehyde-3-phosphate dehydrogenase (Mm99999915_g1) was selected as the housekeeping gene. Relative gene expression was quantified according to the standard-curve method using StepOne version 2.1 (Applied Biosystems).

### Statistical analysis

Significant differences in pregnancy rates, pup birth, and luminal Muc1-positive mice were determined via Chi-square tests, unless expected values were <5. In such cases, Fisher’s exact probability test was used for comparisons. Pup weight, placental weight, number of implantation sites, uterine weight fold-increase, serum progesterone and oestradiol levels, mRNA expression levels, as well as H-scores were all normally distributed. One-way analysis of variance and post-hoc multiple-comparison test were used to determine between-group differences and to separate means. Significance was set at *P* < 0.05.

## Supplementary information

Supplemental Information
