# Supplementary material for: Evaluation of uterine receptivity after gonadotropin releasing hormone agonist administration as an oocyte maturation trigger: a rodent model
Source: Sci Rep. 2019 Aug 29;9:12519. doi: 10.1038/s41598-019-48918-3 (PMC6715633; doi:10.1038/s41598-019-48918-3)

Supplementary information

Evaluation of uterine receptivity after gonadotropin releasing hormone agonist administration as an oocyte maturation trigger: a rodent model

Kenji Ezoe, Nana Murata, Akiko Yabuuchi, Tamotsu Kobayashi, Keiichi Kato*

Kato Ladies Clinic, 7-20-3 Nishishinjuku, Shinjuku-ku, Tokyo 160-0023, Japan

*Correspondence and requests for materials should be addressed to K.K. (email: k-kato@towako.net)

Supplemental Figure 1 Staining intensity criteria for H-score

Supplemental Figure 2 Expression of MUC1 and Ki67 proteins in endometrium on days 1 and 4 of pregnancy. (A) MUC1 distribution. Scale bars represent 100 μm. (B) Proportion of mice positive for luminal MUC1 expression. Values above bars indicate number of MUC1-positive mice over total number of mice. (C) Distribution of Ki67. Scale bars represent 100 μm. (D) H-scores for Ki67 expression. Error bars represent standard error of the mean. Bars labelled with distinct letters are significantly different from each other ($P < 0.05$)

Supplemental Figure 3 Blastocyst implantation and endometrial steroid signalling after LIF treatment or dual trigger. (A) Number of implantation sites on day 5 of pregnancy. Ten blastocysts were transferred to uteri on day 4 of pseudopregnancy, and the number of implantation sites was assessed on day 5. Values above bars indicate the amount of pregnant mice over total number of mice. (B) Distribution of PR in endometrium on day 4. Scale bars represent 100 μm. (C) *Ihh*, (D) *Areg*, and (E) *Gata2* mRNA expression on day 4. Error bars represent standard error of the mean. Bars labelled with distinct letters are significantly different from each other ($P < 0.05$). (F) Distribution of ERα in endometrium on day 4. Scale bars represent 100 μm. (G) *Muc1,* (H) *Ltf*, and (I) *Lif* mRNA expression on day 4. (J) No. of glands per 1 mm^2 of the endometrium on day 4. (K) *Foxa2* mRNA expression on day 4. Error bars represent standard error of the mean. Bars labelled with distinct letters are significantly different from each other ($P < 0.05$). (L) Distribution of phosphorylated STAT3 in endometrium on day 4. Scale bars represent 100 μm.

Supplemental Figure 1

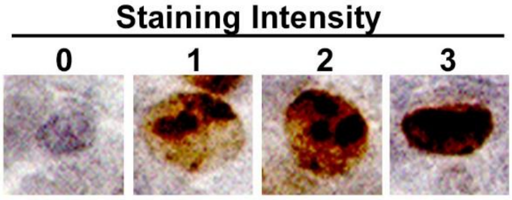

Supplemental Figure 2

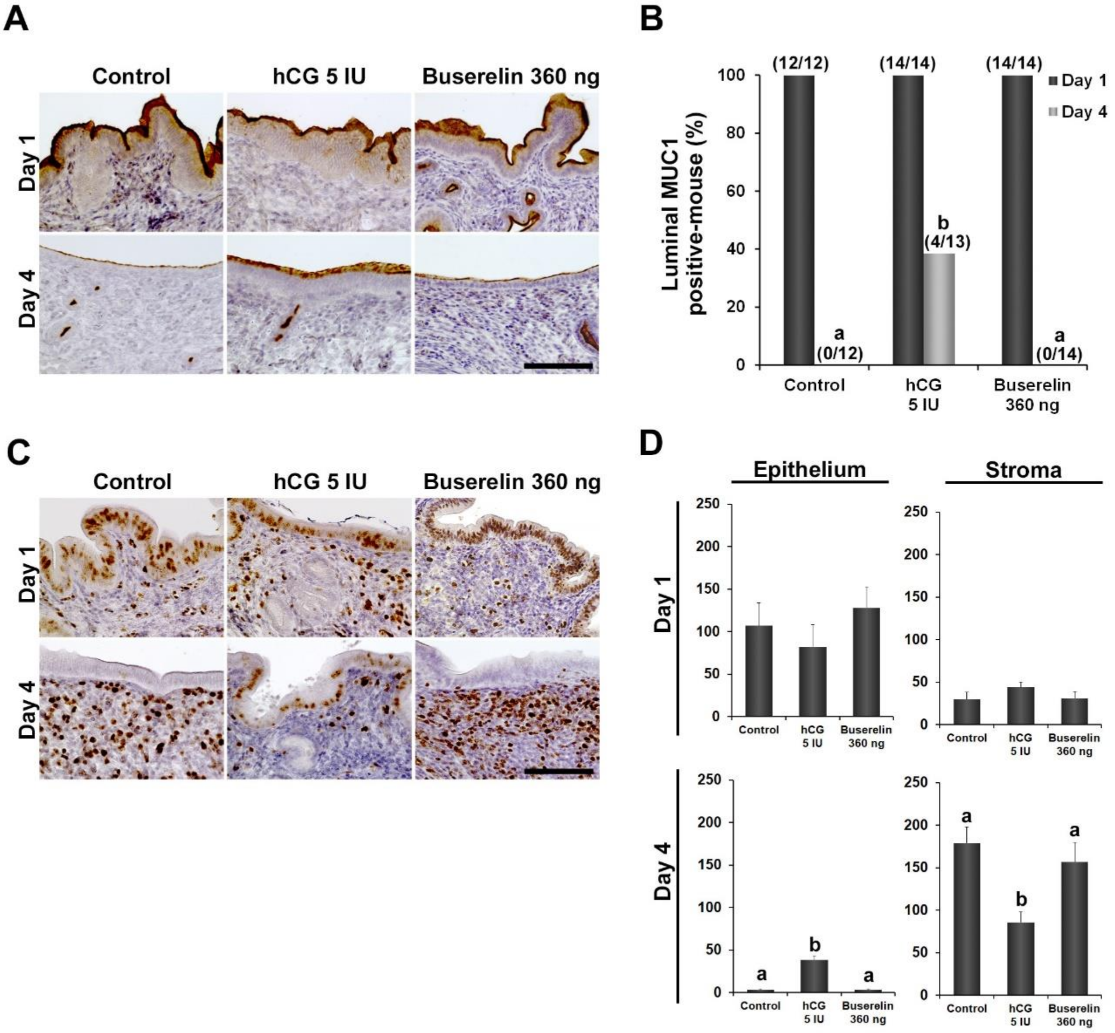

Supplemental Figure 3

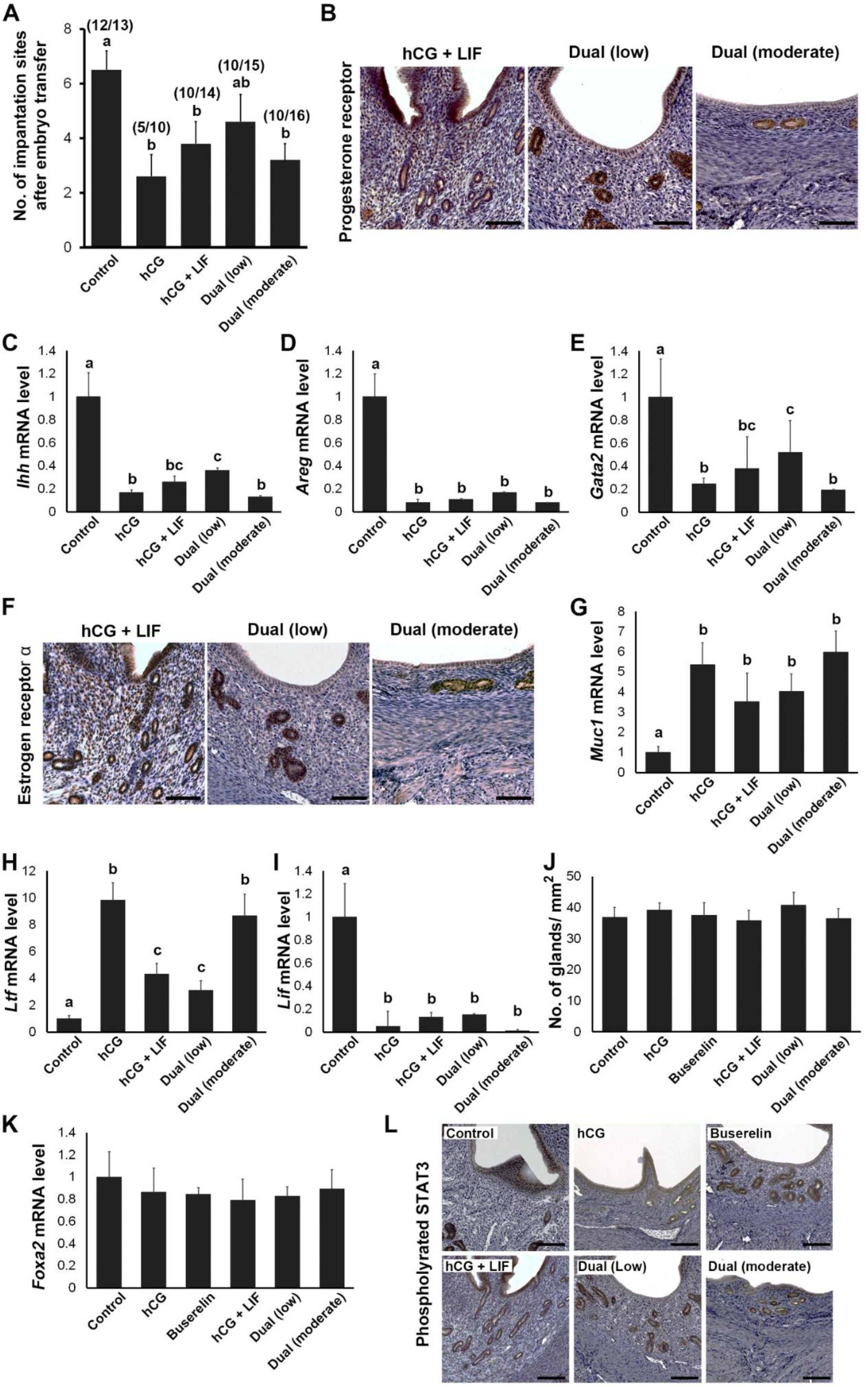

Supplement: Supplementary file 1 — Supplemental Information [file 41598_2019_48918_MOESM1_ESM.pdf]
